# Three-Dimensionally Printed Sensors with Piezo-Actuators and Deep Learning for Biofuel Density and Viscosity Estimation

**DOI:** 10.3390/s26020526

**Published:** 2026-01-13

**Authors:** Víctor Corsino, Víctor Ruiz-Díez, Andrei Braic, José Luis Sánchez-Rojas

**Affiliations:** Microsystems, Actuators and Sensors Group, INAMOL-Universidad de Castilla-La Mancha, 45004 Toledo, Spain; victor.ruiz@uclm.es (V.R.-D.); andrei.braic@uclm.es (A.B.)

**Keywords:** 3D printing, sensor, biofuel, artificial intelligence, optimization, drift

## Abstract

**Highlights:**

**What are the main findings?**

- A novel device integrating 3D-printed sensors with convolutional neural networks for biofuel characterization.
- Development of sensor optimization strategies and spectral data processing techniques.

**What are the implications of the main findings?**

- Successful integration of multiple technologies into a compact, lightweight and highly precise instrument.
- Providing solutions to typical sensor limitations such as resolution, sensitivity and drift.

**Abstract:**

Biofuels have emerged as a promising alternative to conventional fuels, offering improved environmental sustainability. Nevertheless, inadequate control of their physicochemical properties can lead to increased emissions and potential engine damage. Existing methods for regulating these properties depend on costly and sophisticated laboratory equipment, which poses significant challenges for integration into industrial production processes. Three-dimensional printing technology provides a cost-effective alternative to traditional fabrication methods, offering particular benefits for the development of low-cost designs for detecting liquid properties. In this work, we present a sensor system for assessing biofuel solutions. The presented device employs piezoelectric sensors integrated with 3D-printed, liquid-filled cells whose structural design is refined through experimental validation and novel optimization strategies that account for sensitivity, recovery and resolution. This system incorporates discrete electronic circuits and a microcontroller, within which artificial intelligence algorithms are implemented to correlate sensor responses with fluid viscosity and density. The proposed approach achieves calibration and resolution errors as low as 0.99% and $1.48\times 10^{-2}$ mPa·s for viscosity, and 0.0485% and $1.9\times 10^{-4}$ g/mL for density, enabling detection of small compositional variations in biofuels. Additionally, algorithmic methodologies for dimensionality reduction and data treatment are introduced to address temporal drift, enhance sensor lifespan and accelerate data acquisition. The resulting system is compact, precise and applicable to diverse industrial liquids.

## 1. Introduction

The development of sensor devices for controlling the viscosity and density of biofuels has gained considerable importance due to the increasing demand for fuels, their diverse applications and the need to reduce emissions. Although biofuels are generally environmentally favorable, they can lead to elevated emissions or suboptimal engine performance if their viscosity and density are not properly controlled [1,2,3,4]. Numerous studies have explored sensor technologies for monitoring the physical properties of fuels, biofuels and engine oils [3,4,5,6,7,8,9,10,11,12]. More recently, artificial intelligence has become a powerful complement to microsensor technologies, enabling advanced compositional analysis of fuel–lubricant and biofuel mixtures [13,14,15,16]. Nevertheless, many of these approaches rely on expensive instrumentation and complex electronic architectures, which restricts their practical deployment.

To address this limitation, our recent work [17] presented a compact, integrated sensing device. While effective, this design (like other approaches reported previously [4,5,6,7]) relies on conventional fabrication techniques such as lithography and thin-film deposition for sensor production. In contrast, three-dimensional (3D) printing offers a cost-effective and highly precise alternative, positioning it as a promising method for sensor manufacturing. In this context, numerous studies have explored the application of 3D printing technologies in the fabrication of piezoelectric sensors. For instance, in [18,19,20,21,22], the authors employed polyvinylidene fluoride (PVDF) polymers and their copolymers to develop piezoelectric devices for various sensing applications. These include pressure and force sensors tested under controlled laboratory conditions [18,20], acoustic receivers [21], flow sensors for deionized water, mimicking blood flow [19], and tactile sensors embedded in prosthetic hands capable of identifying object hardness [22]. In [23,24], the authors designed micromachined piezoelectric ultrasonic transducers (PMUTs) using PZT sheets mounted on titanium substrates. These were integrated with Helmholtz resonators, featuring 3D-printed cavities of controllable volume to monitor human respiratory patterns. Additional approaches include the use of photopolymer resins mixed with PZT particles to fabricate ultrasonic wave sensors [25], as well as the development of piezoresistive MEMS accelerometers via 3D printing for controlled experimental use [26].

A particularly active area of research involves 3D-printed sensors for liquid analysis, addressed in various studies [27,28,29,30,31]. These works leverage additive manufacturing to produce complete sensor assemblies, fluidic cavities or microfluidic channels. Sensor modalities include substrate-integrated waveguide (SIW)-based designs [27,32,33], microwave resonators [28,30,31,34,35], parallel plate configurations [29], optical interferometers [36], and electromagnetic bandgap (EBG) microstrip sensors [37]. These systems enable the differentiation of liquid compositions by measuring dielectric permittivity or transmission spectra. Applications span petroleum monitoring [35]; aqueous mixtures with water–ethanol blends [27,28,29,30,31] and solutions containing isopropanol [32], glucose [36] or potassium chloride [29]; toluene–methanol mixtures [37]; and various pure chemicals [30,31,33,34,37]. All the aforementioned studies used laboratory instrumentation in their measurement process.

Recent advancements have also integrated machine learning techniques with 3D-printed sensor systems. For example, in [38], the authors developed a deformation-sensitive sensor composed of graphene nanoparticles and carbon nanotubes, employing support vector machines (SVMs) to classify human motion and monitor exhalation; the researchers in [39] applied neural networks to optimize the design of a 3D-printed accelerometer for human movement tracking, while in [40], they fabricated three-axis force sensors and trained a support vector regression (SVR) model on simulated data to quantify forces in medical contexts; and finally, the work in [41] combined multi-sensor 3D printing with machine learning algorithms to predict behavioral impairment following alcohol consumption.

In previous studies such as [42,43], we introduced a 3D-printed sensing cell incorporating piezoelectric actuators, designed to vibrate at a specific resonance, and artificial intelligence (AI) techniques to characterize the physical properties of aqueous glycerin solutions. In the present work, we propose an enhanced version of this type of sensor, designed to broaden its operational bandwidth and improve calibration and resolution results. Furthermore, we integrate the sensor into a microcontroller-based system equipped with convolutional neural networks (CNNs), targeting the application of biofuel characterization.

This article is structured as follows: The Section 2 details the sensor design, geometric parameters, simulation procedures, structural optimization criteria, complete device schematics, experimental methodology, data processing techniques and machine learning algorithms studied. The Section 3 presents optimal sensor geometries identified in real-world applications, experimental data captured with our system from biofuel solutions and the architecture of the most effective AI models, as well as their precision obtained in calibration and from drift experiments, using dimensionality reduction, compared to reference values. In the Section 4, we contextualize our findings within the existing literature and outline potential future research directions. Finally, we conclude with the key contributions and implications of this work.

## 2. Materials and Methods

### 2.1. Comparative Analysis of Sensor Structure and Actuator Layouts

**Figure 1:**
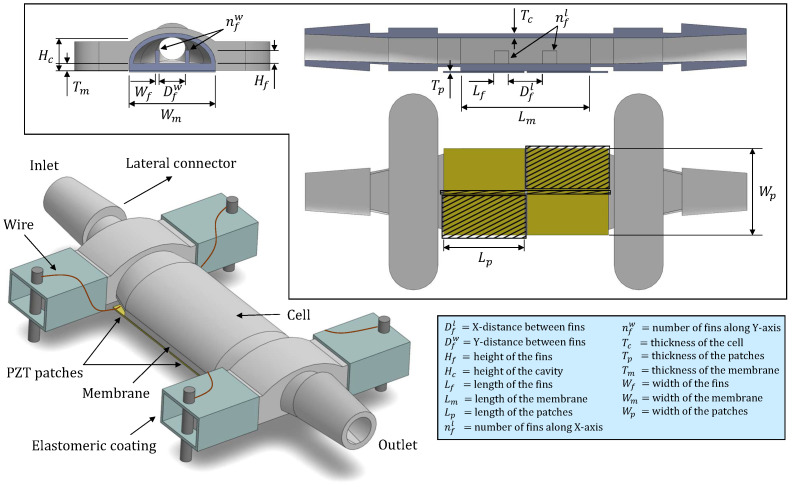
Basic sensor design with the most important geometric parameters. A top view, elevation section and profile section are provided for complete structural characterization.

The fundamental architecture of the developed sensor is illustrated in Figure 1. The device comprises two integrated components, forming a unified system. The first part consists of a semi-cylindrical container cell designed to hold the target liquid. This cell features a vibrating membrane at its base and lateral connectors to facilitate fluid circulation. It was fabricated via stereolithography (SLA) 3D printing technology [44] using a Form3 printer (Formlabs Inc., Somerville, MA, USA) [44] and Rigid10K resin (Formlabs Inc., Somerville, MA, USA) [45], a material validated in previous studies [42,43] for its mechanical robustness (Young’s modulus = 10 GPa). According to manufacturer specifications [45], this resin also demonstrates good chemical resistance against fuel exposure, exhibiting only a 0.1% weight increase after 24 h when a sample structure is immersed in diesel. Key printing parameters, including dimensional tolerance, resolution, printing speed and ultraviolet (UV) light exposure duration, were controlled throughout the manufacturing process. The printed cell was then subjected to an oxygen plasma treatment to improve surface wettability. This process was conducted for 2 min at 50 W plasma power and 0.2 mBar oxygen pressure within a vacuum chamber equipped with a radiofrequency plasma generator (Diener electronic GmbH + Co. KG, Ebhausen, Germany) [46].

The second component comprised the piezoelectric actuators. Commercially available PZT sheets (PIC255, type 5A modified lead zirconate titanate, PI Ceramic GmbH, Lederhose, Germany [47]), 100 microns thick, with top and bottom CuNi metallization and a stiffness of 63 GPa, were manually sectioned and placed on the external surface of the cell membrane using a cyanoacrylate-based contact adhesive Loctite^®^ 401 (Henkel, Düsseldorf, Germany) [48]. This configuration ensured isolation of the piezoelectric ceramics from the diesel medium, preventing potential chemical degradation. The adhesive selection was validated by previous comparative studies, which revealed negligible performance variation across different cyanoacrylate formulations, attributed to the minimal adhesive layer thickness employed and its relatively low stiffness compared to the other structural components of the sensor.

**Figure 2:**
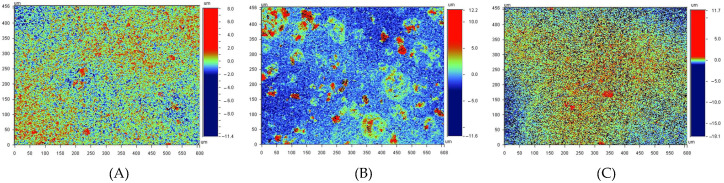
Roughness measurements on 3D-printed resin and PZT films. (**A**) PZT surface 1: $R_{a}\approx$ 650 nm; (**B**) PZT surface 2: $R_{a}\approx$ 1230 nm; (**C**) R10K resin: $R_{a}\approx$ 350 nm.

We assessed the fabrication quality and surface morphology by measuring the roughness of both the 3D-printed part and the PZT films using a Wyko NT1100 optical profiler (VEECO, Plainview, NY, USA) [49]. The results are presented in Figure 2. The gap between the PZT and resin was filled with adhesive, forming an ideally uniform and elastic multilayer. Regarding the resin surface in contact with the liquid, its roughness may affect resonance behavior depending on its relative magnitude compared to the acoustic boundary layer thickness, as discussed in [50]. For diesel fuel, the acoustic boundary layer thickness across the relevant resonance range (10–600 kHz) varies from approximately 10 μm to 1 μm. Although the resin surface can be considered relatively smooth compared to this scale, minor effects were expected and considered part of the sensing capability of the devices.

To establish electrical connectivity, 100 μm Cu wires were soldered to each end of the piezoelectric patches. The opposite ends of the wires were connected to pins embedded in elastomeric plastic tubing for mechanical isolation. The integrity of the solder joints was verified by analyzing the impedance spectrum of the patches across a frequency range up to 1 MHz using an Agilent^®^ 4294A impedance analyzer (Keysight, Santa Rosa, CA, USA) [51]. These elastomer-coated pins were subsequently inserted into the cell’s side connector protrusions, yielding a compact assembly and minimizing the influence of unwanted variable mechanical coupling caused by the introduction of the cell into a ZIF socket interface for electronic connection. While fabrication tolerances may influence the final electromechanical response, each device was individually characterized and calibrated.

The sensors manufactured following the described procedure operate as follows: Excitation of the actuation piezoelectric plates by a periodic signal induces structural vibrations. These perturbations are modulated by the physical properties of the surrounding fluid [52,53], as density contributes to added mass while viscosity increases damping. Consequently, the spectra read at the detection ports exhibit shifts in their resonant frequencies depending on the density variations between different liquids. Moreover, changes in viscosity modify the quality factor of the peaks, and therefore their amplitude and bandwidth. By applying machine learning techniques, correlations between these vibrational responses and the fluid properties can be systematically established.

An additional key consideration is the structural optimization aimed at achieving multimodal responses. This process was divided into two primary components: the piezoelectric plates and the fluidic cell. The optimization of patch configurations was assisted by finite element simulations in COMSOL^®^ Multiphysics 6.3 [54], enabling a comparative analysis of different actuator layouts based on their spectral response. In contrast, the design of the fluidic cell geometry was addressed experimentally due to the complexity of incorporating full three-dimensional fluid–structure interaction in the model. Multiple prototypes were fabricated and tested under controlled conditions, applying a performance metric focused on resonance amplitude, repeatability and sensitivity to fluid changes to identify the most suitable architectures.

**Figure 3:**
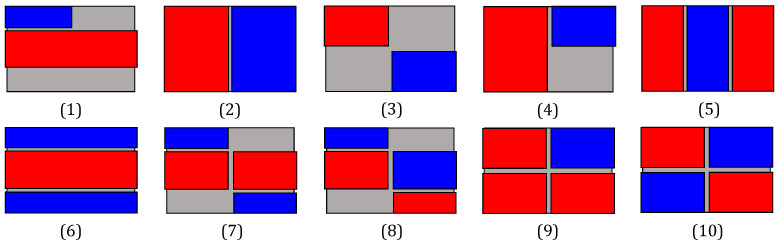
Simulated membranes with piezoelectric patches for actuation (depicted in red) and signal detection (shown in blue).

**Figure 4:**
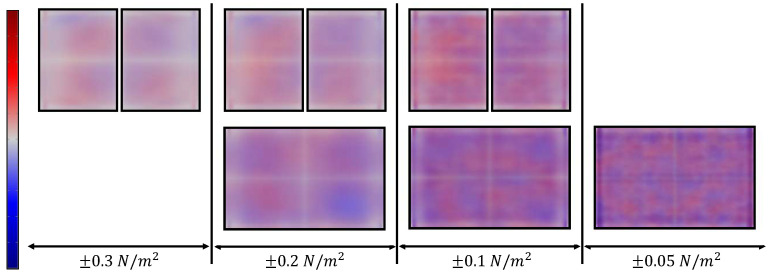
In-plane stress distribution, averaged across all modal forms between 1 kHz and 1 MHz. Positive values are represented in red, negative values in blue and low-stress regions in white. Sensor patches are delineated in black.

The geometry and quantity of piezoelectric films play a critical role in sensor design, as these components, being the most rigid elements, exert a dominant influence on the device’s response. Although simultaneous optimization of the host structure and patch geometry could be addressed as described in [55], this work focuses on a simpler approach: a computational evaluation comparing various actuator configurations to identify the optimal designs. To facilitate this analysis, a simplified cell model was developed using COMSOL. Mechanical and electrical boundary conditions were accurately defined, while non-essential geometric features such as side connectors were excluded due to their lesser impact on the sensor’s dynamic behavior. A mesh-convergence study was performed to ensure that the finite element results were independent of mesh density while maintaining computational efficiency. Additionally, fluid–structure interactions were omitted to streamline the simulation within a three-dimensional framework. Figure 3 illustrates the piezoelectric patch configurations analyzed using this approach. Performance assessment was based on the voltage amplitude spectrum within the frequency range of 1 kHz to 1 MHz. The optimization criterion aimed to maximize both the number and amplitude of resonance peaks in the spectra as these serve as sources of sensor data related to the target liquid [56]. The simulation results indicated that geometries 1 and 6–8 yielded suboptimal spectral profiles. Complementing this analysis, we conducted natural frequency simulations of the standard cell model either with a single piezoelectric sheet on its membrane or with two patches aligned along the vertical axis. In-plane stress values were averaged across all modal shapes to identify regions of maximum and minimum mechanical deformation, thereby revealing zones of enhanced piezoelectric coupling efficiency and enabling the strategic placement of structural elements to support multiple modal responses. The resulting distribution, shown in Figure 4, demonstrates that the superposition of modes within the studied frequency range generated low-stress regions at the mid-width and mid-length of the structures, while concentrating effective actuation and sensing areas in the quadrants. This observation justifies the placement of patches according to the proposed arrangement (configurations 3, 9 and 10), further reinforcing previous conclusions. Based on the combined outcomes of both studies, fabrication efforts were focused on configurations 2–4 and 9–10, which are also depicted in the sensor schematic in Figure 1, with the possible cutouts of actuators highlighted by a black mesh in the plan view.

The other key component is the fluidic cell. As previously outlined, one objective of this study was to develop a sensor capable of producing spectra characterized by a high number of resonances with high amplitude in a certain frequency range. However, these spectral features must also exhibit sensitivity to fluid changes, maintain repeatability across measurements to attain good resolution and demonstrate recovery upon reintroduction of the reference fluid. To achieve this, we experimented with the geometric parameters depicted in Figure 1. Sensors were fabricated using parameter combinations within the ranges specified in Table 1. Iterative adjustments were made based on comparative performance, with superior parameter values informing the design of subsequent prototypes. This approach enabled progressive refinement of sensor architecture.

The method for optimizing the cell structure consisted of the following experimental procedure. A sensor fabricated with a given set of geometric parameters was subjected to two fluids via a peristaltic pump: one comprising pure diesel fuel, and the other consisting of a 90:10 volumetric mixture of diesel and commercial sunflower oil. Starting with pure diesel, spectral data were acquired using an Analog Discovery 2 instrument (Digilent co NI, Austin, TX, USA) [57] across a frequency range of 1 kHz to 1 MHz, discretized into 800 points. A sinusoidal input signal of 3.3 V was applied, preceded by a brief stabilization period to ensure fluid equilibrium. For each frequency point, measurements were performed over at least 10 signal periods and were averaged to improve data robustness. Two spectra were recorded with a time interval of 5 min between successive acquisitions. Subsequently, the pure diesel was replaced with the adulterated mixture, using no further cleaning other than the entrainment effect of the new solution. The same measurement protocol was repeated, and finally, the system was flushed with pure diesel once more, and the measurement cycle was repeated. All measurements were conducted under static fluid conditions at room temperature (22 ± 1 °C).

This experimental framework enables the evaluation of three key performance metrics: sensitivity, reproducibility and recovery. These were quantified as absolute differences between spectra of different solutions ($sensitivity=|s_{j}^{i}-s_{j+1}^{i}|$); spectra of different consecutive measurements corresponding to the same solution ($resolution=|s_{j}^{i}-s_{j}^{i+1}|$); and spectra of the same fluid measured before and after exposure to a different fluid ($recovery=|s_{j}^{i}-s_{j}^{\prime }i|$). The expression $s_{j}^{i}$ denotes the spectrum corresponding to measurement *i* of liquid *j* (either pure diesel or the adulterated mixture). In our case, the index *i* was restricted to {1,2}, as only two resolution measurements were conducted at each step, thereby conditioning the mathematical definition of the three key metrics. Nevertheless, additional measurements could be incorporated with the same definitions applied iteratively and considering either the maximum or the average across operations. Furthermore, the inclusion of the absolute value in the metric expressions ensures insensitivity to the order in which the two liquids are compared. A higher sensitivity is desirable, indicating strong differentiation between fluid types, whereas elevated differentiation in resolution or recovery is undesirable, as it reflects inconsistency in repeated measurements of the same fluid.

Based on these definitions, to determine whether greater sensitivity ‘compensates’ for poorer resolution and recovery at each spectral point *i*, we propose the following equation. The following formulation also serves as a dimensionality reduction strategy for subsequent analysis:

(1) $$m_{i}={\left[\frac{sensitivity}{\max_{diff}\left(resolution,recovery\right)}\right]}^{p}$$

where “max” denotes the greatest difference, corresponding to the worst result between resolution and recovery. To quantify the relative performance of each cell, two metrics are proposed: the first uses Equation (1) to compute *m* over all points of the spectrum and p=2, while the second uses only the points where sensitivity compensates for resolution/recovery (m>1) and p=1. Then, in both cases, the mean value across all spectral points is calculated and sensors with higher values of *m* are considered the best.

### 2.2. System Description and Calibration–Drift Experiments

Here we present the architecture of the measurement system designed to operate the sensors optimized using the method described in the preceding subsection, together with the experimental protocol implemented for calibration and drift assessment.

**Figure 5:**
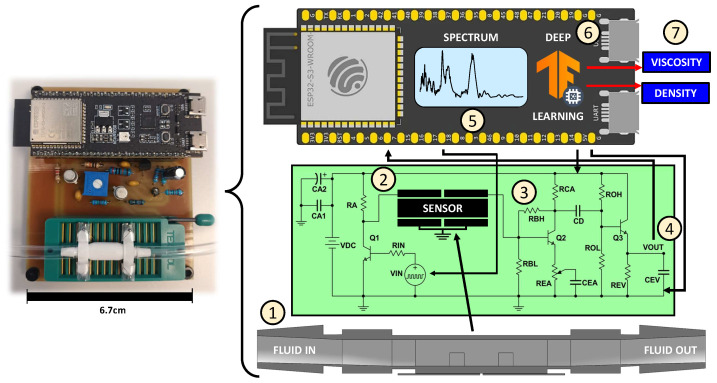
Architecture of our device (sensor + electronics + MCU). The measurement workflow is as follows: (1) liquid insertion, (2) sensor excitation through amplified PWM signal, (3) output signal amplification, (4) envelope reading by A/D converter, (5) repetition for each frequency to obtain a spectrum, (6) data processing and analysis using AI and (7) estimation of liquid viscosity and density.

The highest-performing sensors were integrated into a measurement system analogous to that depicted in Figure 5, previously introduced in [56] for MEMS-based detection of adulterants in olive oil. In addition to the sensor element, the system comprised electronic interfacing circuits designed to operate with an ESP32-S3-DevKitC-1-N32R8V microcontroller (Espressif Systems Co., Ltd., Shanghai, China) [58]. Fluid samples were introduced into the sensor at their maximum flow rate using a Minipuls 3 peristaltic pump (Gilson Inc., Middleton, WI, USA) [59]. A transistor-based circuit was employed to amplify the Pulse Width Modulation (PWM) signal generated by the microcontroller, which served to excite the sensor’s input actuator patch(es). The PWM frequency was swept from 0.05 to 1 MHz, yielding 530 discrete frequency spectra. A variable frequency step was used to account for the microcontroller’s limited frequency resolution. The sensor’s output signal was further amplified via a bipolar junction transistor (BJT) biasing circuit incorporating emitter and collector feedback to improve DC stability at the operating point. This amplified signal was processed by an envelope detector, also implemented using a discrete transistor circuit, and captured via the microcontroller’s analog-to-digital converter (ADC). For each excitation frequency, 1000 output samples were acquired, each comprising 10 response periods. Averaging across these samples improved data robustness. All electronic components were powered using the 5 V supply provided by the microcontroller.

The measurement procedure was repeated for each of the biodiesel solutions listed in Table 2. These solutions were prepared by blending commercial diesel fuel with sunflower oil in proportions consistent with those reported in the literature and compliant with European regulatory standards [2]. This table contains the physical properties of the fluids, determined by averaging three measurements obtained using a commercial DMA4100M densitometer equipped with a Lovis module (Anton Paar GmbH, Graz, Austria) [60], maintained at 40 °C. We can observe that both viscosity and density increase nearly monotonically with oil content, although with distinct slopes, thereby supporting their treatment as independent variables in AI models. Even minor compositional changes in certain mixtures lead to measurable variations in viscosity and/or density, influenced by factors such as mixture tolerances, temperature effects and the non-linear scaling of these properties with oil percentage at low adulteration levels.

Samples were introduced into the system in ascending order of viscosity. For each fluid, measurements were conducted under static conditions following a stabilization period of 5–15 min, and subsequently, 33 spectra were collected over approximately 10 min. When changing the fluid, the sensor was flushed with the incoming fluid for 3 min without any additional cleaning protocol. All measurements were performed without active temperature control of the resonator; the ambient temperature during the experiments was maintained at 23 ± 1 °C. The measurement system operated in real time and did not require opening of the fluidic circuit. We experimented by acquiring measurements under both continuous-flow and static conditions and examining the evolution of the spectral response across consecutive acquisitions, which exhibited reduced variability when the fluid was at rest. Furthermore, the experiment was repeated using solutions $N_{2}$, $N_{3}$, $T_{3}$ and $T_{4}$ over a period of four days to assess whether spectral variations arising from short-term temporal drift could be mitigated through data processing and AI techniques.

### 2.3. Deep Learning Algorithm Optimization

Following the acquisition of spectral data from all the prepared solutions, machine learning models were constructed to establish predictive relationships between the spectra and the corresponding physical properties of the fluids. As demonstrated in our previous studies [17,43,56], convolutional neural networks exhibited strong performance in the analysis and interpretation of spectral signals. The procedures employed to identify network architectures best suited to our data, together with the spectral drift compensation and dimensionality reduction strategies implemented, are detailed below.

The first step involved defining the data sets and applying appropriate scaling. Mixtures labeled $N_{1}-N_{5}$ were designated as the training set, whereas mixtures labeled $T_{1}-T_{6}$ were reserved exclusively for testing and were not used during parameter optimization. Prior to training, the data were preprocessed using a scaling method based on the standard deviation. Following this process, optimization of the neural network architecture was undertaken. To identify the best network configurations that improve predictive accuracy, we applied a hyperparameter fine-tuning strategy analogous to that used in [17,56]. Specifically, we varied the number of convolutional layers (up to five), the number of filters, their size and their stride. The tested parameter values are illustrated in Table 3, which summarizes the general architecture of the evaluated models. The filters and their associated parameters define distinct mechanisms for spectral feature extraction. The ReLU activation function was employed to facilitate gradient-based optimization via the Adam algorithm [61], which was proven effective in CNN training. Learning rates ranging from 0.01 to 0.001 were explored to minimize the mean squared error loss function. To control model complexity, we incorporated either high stride values or max-pooling layers, which performed local subsampling by selecting the maximum value within a defined neighborhood. Following the final convolutional block, an average pooling layer was applied to reduce dimensionality, thereby removing the need for a flattening operation and fully connected layers. This design choice was critical, as the latter approach introduces additional parameters and computational overhead, which are incompatible with the constraints of microcontroller deployment. The network ended with a single output neuron using a linear activation function to predict the target physical properties, such as viscosity and density. To identify the most effective model architecture, a comparative evaluation was conducted by quantifying the prediction error. This error was computed as the arithmetic mean of the absolute differences between the model-predicted values of viscosity or density and the corresponding reference values obtained from the commercial laboratory-grade viscometer. This approach enabled the selection of the network configuration that yielded the highest predictive accuracy. All models were implemented and trained using TensorFlow 2.17 [62].

The models were subsequently evaluated using data obtained from drift experiments. To simulate realistic operating conditions, liquid $T_{3}$ was selected as the reference fluid for measurements on different days. We employed this liquid as a reference because it constitutes a representative solution of the problem under study, namely biodiesels derived from mixtures of conventional diesel and sunflower oil, and it exhibits a comparatively low viscosity relative to other biofuels in our dataset, thereby enabling more efficient removal during the circulation of subsequent liquids to be measured. Following the measurement protocol described earlier, the mean spectrum was calculated from repeated measurements. The ratio between the average spectrum of the reference solution on the initial day (day 0) and that obtained on day *n* was then determined. This ratio defines a spectral factor $r_{n}$, which encodes the relative variations in sensor response between day 0 and day *n* across all spectral components, each of which may evolve differently over time. The factor $r_{n}$ was then employed to adjust the spectra of subsequent solutions. Specifically, the adjustment consisted of multiplying the measured spectrum of a given solution by $r_{n}$, thereby approximating a transformation of the data from day *n* to day 0. The corrected spectrum was then reintroduced as input to the trained machine learning models, yielding more accurate predictions of viscosity and density compared to unprocessed spectra. In this way, the operational lifespan of both the sensor and the overall system can be extended simply by using one single unique calibration solution as reference.

In addition, a dimensionality reduction study was conducted as follows. Using Equation (1) as a basis, the number of spectral points was progressively reduced, thereby decreasing measurement time while simultaneously evaluating the impact on neural network training and performance in both calibration and drift experiments.

It is important to note that the models were trained externally and subsequently deployed on the microcontroller via the TensorFlow Lite library, enabling a fully autonomous viscosity and density estimation system. This machine learning framework not only enhanced system autonomy but also simplified the electronic requirements for signal processing because any spurious effect originating from the parasitic elements of the resonator or the electronics remained constant across all measurements, so they could be learned and disregarded by the machine learning models.

## 3. Results

This section applies the previously defined performance metric to evaluate and compare 64 sensor geometries fabricated during the design refinement process. It presents representative examples illustrating how the criterion guided structural selection, summarizes the final geometric parameters and discusses the influence of piezoelectric patch distribution and fluidic cell features on overall sensor performance.

In addition, we describe the outcomes of the calibration and drift experiments performed with the optimized sensor integrated into the microcontroller-based system. These results include spectral measurements for all prepared solutions, the predictive performance of the CNN models for viscosity and density estimation, and their comparison with laboratory references. This section also examines the correction of temporal drift and explores dimensionality reduction as a means to accelerate measurements while preserving accuracy and enabling efficient deployment on resource-constrained hardware.

### 3.1. Optimization of the Sensor Structure

**Figure 6:**
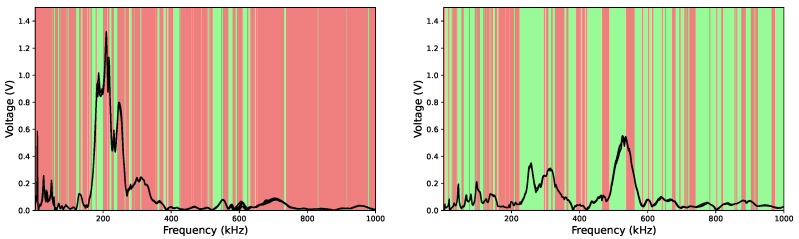
Comparison of the performance of two geometries: a cell with poorer *m* and performance in the sensitivity reproducibility/recovery game (**left**), and a sensor with improved performance across this metric (**right**). The black curves correspond to the spectra measured with these devices, while the green and red lines emphasize the components with m>1 and m<1, respectively.

As described in Section 2, we employed a systematic approach to refine the geometry of the liquid-containing cell, guided by Equation (1) and the measurement protocol described in the corresponding subsection. According to this equation, spectral points yielding values greater than 1 are considered positive, as they indicate that the sensor’s sensitivity exceeds its limitations in resolution or recovery. Conversely, values below 1 are regarded as negative, since insufficient resolution or recovery cannot be distinguished from spectral variations induced by changes in the liquid. In total, 64 combinations of geometric parameters were designed, fabricated and tested. Figure 6 illustrates the application of the optimization method to two representative geometries: one of larger dimensions without fins (left), and another of a smaller scale incorporating membrane extensions (right). In the following figure, green lines indicate cases where m>1, while red lines correspond to m<1, enabling refinement of the cell structure based on this criterion.

**Figure 7:**
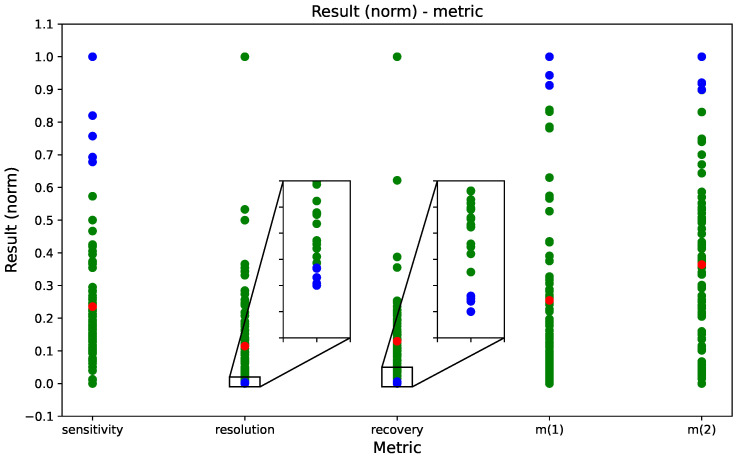
Normalized values of sensitivity, resolution, recovery and *m*-based metrics ($m_{1}$ and $m_{2}$) for all geometric parameter combinations evaluated (shown as green points). The mean of each metric is indicated in red, and the five highest-performing results are highlighted in blue.

Figure 7 presents the normalized results for sensitivity, resolution, recovery and the two measurements based on the *m* metric ($m_{1}$: computed using all spectral points with p=2; and $m_{2}$: computed using only the points for which sensitivity compensates for resolution/recovery, i.e., m>1, with p=1) for the different sensor geometries evaluated. Higher sensitivity and *m* values, together with lower resolution and recovery errors, indicate superior performance. The five best-performing geometries for each metric are highlighted in blue in Figure 7, and Table 4 summarizes the geometric parameter ranges associated with these top-ranked designs. From these results, the following conclusions can be drawn:

- In general, miniaturization of the cell and its patches yielded improved resolution and recovery, resulting in higher *m* metric values.
- When the cell membrane was fully covered by piezoelectric actuators, the addition of fins produced inferior results compared to an empty configuration. Conversely, when the actuators were reduced in size such that half of the membrane remained uncovered, the presence of fins substantially enhanced the *m* metrics relative to a hollow cell.
- A comparable improvement was observed when the membrane thickness was reduced. However, combining fin addition with reduced membrane thickness led to overall poorer performance, as each factor independently increased variability in the printing process, and their simultaneous application amplified this variability. Accordingly, a thicker membrane proved more suitable when fins were incorporated, providing leverage between peak amplitude and manufacturing resolution.
- The optimal fin configuration consisted of one fin per quadrant of the cell (i.e., a 2 × 2 array). Increasing the number of cilia along either axis degraded performance.
- A reduced arrangement (1 × 2) produced comparable results only when the cilia were positioned within quadrants.
- Theoretically, a greater height of the fins increases fluid–structure interaction but maintains a free end so they can move more freely.
- While complete coverage of the membrane with four piezoelectric patches increases sensitivity, it simultaneously degrades resolution and recovery, significantly worsening the *m* metric values, likely due to multiplied defects introduced during fabrication and gluing of the patches.

It should be emphasized that these conclusions are constrained by inherent imperfections in the sensor’s fabrication. Manufacturing processes introduce additional sources of variability, including cell curing, resin evacuation, patch cutting and non-uniform adhesion. Therefore, the findings presented here should be regarded as design guidelines rather than definitive optimization criteria.

**Figure 8:**
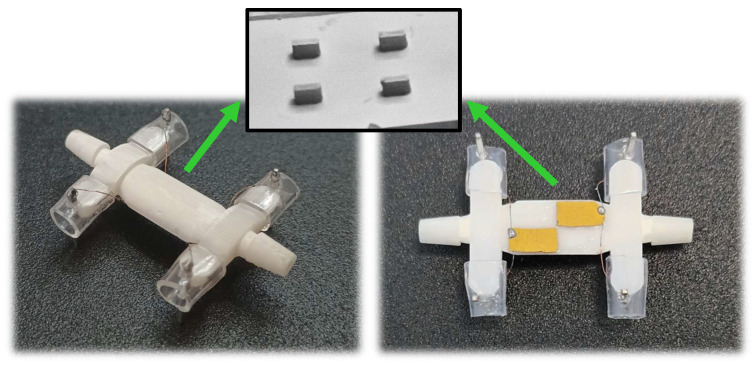
Sensor obtained through the structural refinement process. Three views are presented: an overview (**left**), a plan view (**right**) and an interior view of the cell showing the arrangement of fins attached to the membrane (**top center**).

Figure 8 and Table 4, “best” present the outcomes of these structural refinement techniques. Regarding patch components, both vertical and horizontal divisions were observed, as described earlier. For the fluidic cell, a compact design incorporating four internal fins, one per rectangular quadrant, proved sufficient to achieve good results across the three defined metrics while minimizing material requirements. Notably, alternative models based on this geometry, such as those with a 0.2 mm membrane or without fins, also performed well experimentally. These designs also expanded the range of significant frequencies relative to our previous works [42,43] and increased the freedom of vibration, resulting in a more informative spectral response.

### 3.2. Calibration and Drift Experiments

**Figure 9:**
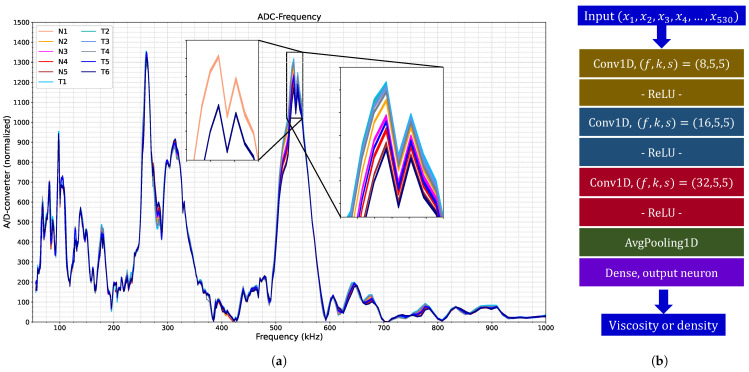
(**a**) Spectra collected by our system for each solution in Table 2. (**b**) Optimized neural network architecture for viscosity and density estimation based on the *N*-labeled spectra in (**a**).

The sensor depicted in Figure 8 was integrated into the microcontroller system illustrated in Figure 5. Subsequently, experiments were conducted to measure the solutions listed in Table 2, following the protocol described in Section 2.2. A total of 363 spectra were acquired, as shown in Figure 9a. The spectra exhibit several resonance peaks that are sensitive to variations in fluid properties. As discussed earlier, progressive increases in viscosity and density alter the damping and added mass, respectively, leading to gradual modifications in both the amplitude and shape of these resonances and thereby improving spectral separability between solutions. The zoomed-out view on the left of the figure illustrates how one of these peaks changes for two liquids with markedly different viscosities. Notably, viscosity and density perturb each peak in distinct ways, resulting in complex spectral behavior that conventional analytical methods cannot adequately capture. In contrast, CNNs statistically integrate all these coupled physical effects by leveraging the full multimodal spectrum, enabling a more comprehensive and robust interpretation of the sensor response.

Based on these data, convolutional neural networks were trained to predict viscosity and density using the optimization procedure explained in Section 2.3. The solutions labeled *N* in Table 2 served as training data, while the remaining fluid measurements were reserved for testing, thereby assessing the model’s generalization performance. The optimal network architectures identified for both viscosity and density estimation follows the configuration shown in Figure 9b. This architecture comprises three convolutional layers with 8, 16 and 32 filters, respectively, each with a kernel size of 5 and a stride of 5, without intermediate max-pooling layers. The network concludes with a global average pooling layer and a dense output layer containing a single neuron, and training was performed with a batch size of 55 over 200 epochs. The models occupied 500 kB, using 41% of the microcontroller’s program storage space. The measurement time for a complete spectrum was 16 s, but once taken, the estimation of the physical properties models was completed in milliseconds.

**Figure 10:**
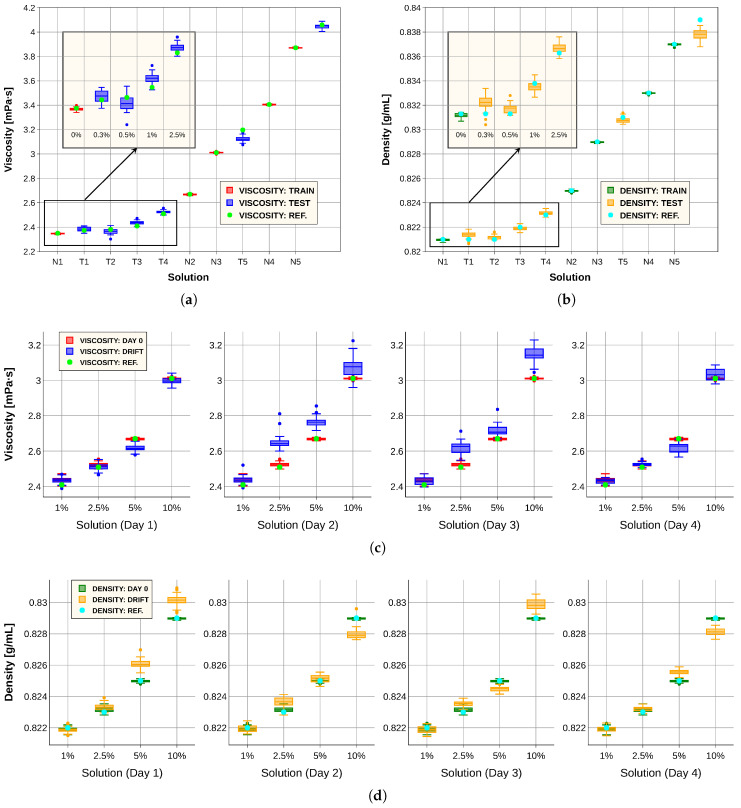
(**a**) Viscosity model predictions for the training set (red) and test set (blue), compared with measurements from the commercial viscometer (green). (**b**) Density model predictions for the training set (green) and test set (orange), compared with the reference measurements (blue). (**c**) Viscosity predictions for four solutions at day 0 (red) and over four subsequent days after applying the data correction procedure (blue), compared with the reference values (green). (**d**) Density predictions for four solutions at day 0 (green) and over four subsequent days after applying the data correction procedure (orange), compared with the reference values (blue).

Figure 10a,b present the results obtained with our predictive models in comparison with the laboratory instrument. In Figure 10a, the red boxes denote viscosity predictions for the training solutions (*N*), the blue boxes correspond to the test solutions (*T*) and the green dots indicate the reference values measured with the commercial viscometer. Similarly, Figure 10b shows the density predictions, where the green boxes represent the training set, the orange boxes the test set and the blue dots the reference measurements. For viscosity, the system successfully distinguished between pure diesel and a mixture containing 1% sunflower oil; however, minor adulterations remained undetectable, as evidenced by the overlap of boxplot extremes. The density predictions were also very accurate.

To further evaluate system robustness, we repeated the measurement procedure described in Section 2.2 for fluids $N_{2}$, $N_{3}$, $T_{3}$ and $T_{4}$ over four consecutive days. This experiment enabled the development of an algorithmic approach to extend sensor lifespan. Specifically, the previously trained models were combined with the drift correction method explained in Section 2.3, yielding the results shown in Figure 10c,d. These figures compare predictions obtained from spectra recorded on day 0, corresponding to the initial measurement of the 11 solutions, with those derived from spectra collected on subsequent days. Spectral variations attributable to temporal drift were observed, underscoring the need for correction. Although prediction accuracy decreased over time, the integration of the models with the spectral transformation method still allowed differentiation among most solutions. This demonstrates that simple algorithmic strategies can effectively mitigate drift effects and extend the operational lifetime of the sensing system, achieving accuracies sufficient for certain practical applications.

Table 5 and Table 6 report the mean errors associated with the viscosity and density models during the calibration experiment, whereas Table 7 and Table 8 present the corresponding results for the drift experiment. Consistent with previous studies [56], several metrics were employed to evaluate model performance: the absolute difference between the predicted and reference values (*e* (mPa·s) and *e* (g/mL)); the ratio between the mean error and the operating range (full-scale error, *e* (%FS)); the relative deviation, defined as the mean difference normalized by the reference values (*e* (%RD)); and the resolution error, quantified as the standard deviation across repeated measurements of the same solution ($e_{res}$). These metrics effectively implement a minimum detectable change in density and viscosity. The tabulated results corroborate the conclusions observed in the graphics: both calibration and resolution errors are minimal, and when comparing the drift experiment outcomes with those obtained on day 0, the errors increase slightly but remain within acceptable limits.

**Figure 11:**
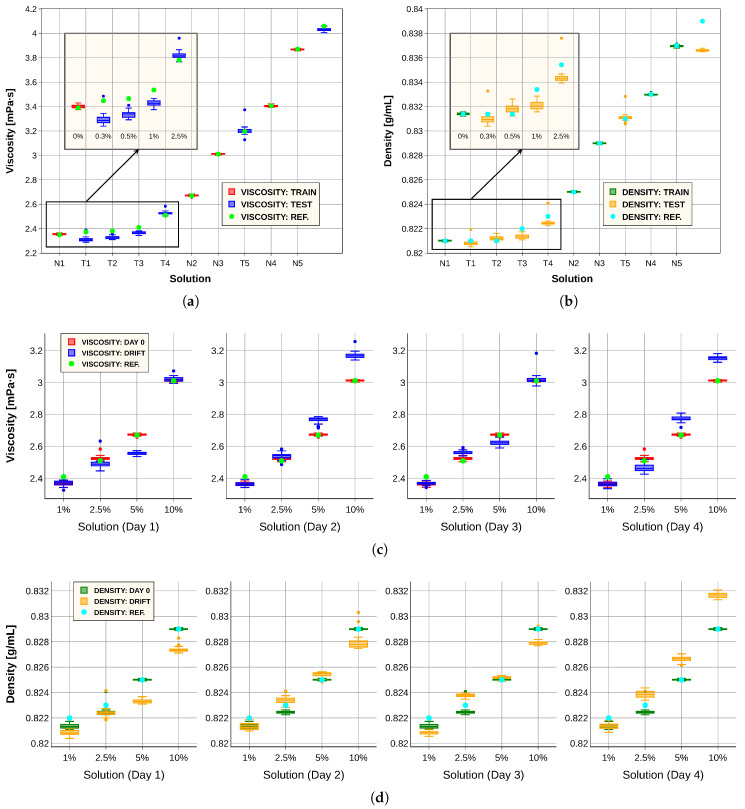
Results obtained using models trained with 103-point spectra. (**a**) Viscosity model predictions for the training set (red) and test set (blue), compared with measurements from the commercial viscometer (green). (**b**) Density model predictions for the training set (green) and test set (orange), compared with the reference measurements (blue). (**c**) Viscosity predictions for four solutions at day 0 (red) and over four subsequent days after applying the data correction procedure (blue), compared with the reference values (green). (**d**) Density predictions for four solutions at day 0 (green) and over four subsequent days after applying the data correction procedure (orange), compared with the reference values (blue).

Finally, a dimensionality reduction analysis was performed using metric Equation (1) and the new definition of resolution as the standard deviation, with the objective of reducing data acquisition time and thereby accelerating the estimation of fluid physical properties. To this end, progressively fewer spectral points were selected according to Equation (1), and new convolutional neural networks were trained following the protocol described in Section 2.3. The trained models were applied both to the calibration experiment and to the drift experiment. Table 9, Table 10, Table 11 and Table 12 summarize the errors obtained. The results indicate that performance remains comparable whether 530 or 103 spectral points are used, demonstrating that measurement time can be reduced with a minimal impact on accuracy. Moreover, reducing the input dimensionality to 103 points lowered computational demands; that is, the optimal neural networks contained fewer parameters, improving their suitability for implementation in the limited memory of the microcontrollers. Figure 11 presents the graphical results obtained with models trained on spectra of 103 points, confirming their strong overall performance when compared with models trained on spectra of 530 points (Figure 10).

## 4. Discussion

Using the error values reported in the previous section, the performance of the proposed device can be compared with other viscosity and density measurement systems described in the literature [5,6,7,9,10,17,42,43]. The results of this comparison are presented in Table 13 and Table 14. For the viscosity measurements, the proposed system achieves the second-best performance in the *e* (mPa·s) and *e* (%FS) metrics, ranking only behind [17], while in the *e* (%RD) metric, it occupies the third position (after [7,17]). In terms of density, the accuracy of our system consistently ranks third across the *e* (g/mL), *e* (%FS) and *e* (%RD) metrics. With respect to resolution, the device ranks close to fourth, corresponding to the results reported in [17]. It should be emphasized that the studies compiled here are not restricted to fuels; they also encompass other types of liquids, whose interactions with the sensor material may yield either improved or diminished performance. A particularly relevant comparison can be observed in the column corresponding to [17]. In their case, the reported errors were obtained using a similar system configuration and a comparable set of biofuels but employing a MEMS sensor. The results were nearly identical, although in their study, it was possible to detect a 0.5% admixture of sunflower oil in their diesel. These findings highlight that a sufficiently precise system can be realized using low-cost technologies, especially when combined with artificial intelligence, thereby achieving performance comparable to sensors fabricated with more expensive technologies.

In this work, we introduced optimization strategies for multiresonance analysis, integrated 3D-printed sensors into a microcontroller-based platform and addressed previously unexplored challenges related to dimensionality reduction and drift correction. Building on our earlier contributions, we demonstrated that 3D-printed sensors can achieve performance comparable to MEMS-based devices for biofuel property detection when combined with artificial intelligence. Despite these advances, several avenues for further development and system optimization remain.

First, although the PZT elements employed in this study were encapsulated and isolated from the fluid, being bonded to the external surface of the membrane inside the fluidic chamber, future designs would benefit from the use of lead-free piezoelectric materials. Lead-free ceramics such as (K,Na)NbO_3_ (KNN) [63] and Bi_0.5_Na_0.5_TiO_3_ (BNT) [64] have emerged as promising alternatives to conventional PZT due to environmental and regulatory constraints associated with lead, and recent material improvements further support their suitability as replacements.

Second, enhancements to the fabrication process are needed. Two potential strategies include the following: (i) laser-cutting the piezoelectric patches to improve sample-to-sample reproducibility, followed by bonding using the printing resin, or (ii) incorporating piezoelectric particles directly into the polymer resins, thereby producing a fluidic cell with spatially uniform sensitivity. Although both approaches could improve manufacturing consistency, the intrinsic resolution limits of the 3D-printing process would remain a constraint.

The experiments conducted here relied on discrete, pre-prepared mixtures under controlled laboratory conditions, which may limit direct applicability to continuous online industrial monitoring. Future improvements could therefore involve the use of materials with reduced chemical reactivity toward fuels, enabling greater long-term stability and allowing the system to exploit its potential capability for continuous operation (an aspect not used in the present study, where measurements were performed with the liquid at rest). Additional target liquids could also be explored. Since sunflower oil was considered exclusively as a biofuel additive, it would be valuable to assess the system’s robustness against common interferences such as water contamination, oxidation products or dissolved gases, particularly when these factors induce density or viscosity variations beyond the resolution limits of the sensor. Another promising direction involves evaluating temperature effects and implementing compensation strategies, either electronically or by incorporating temperature as an additional input during neural network training.

The proposed system relies on deep learning to map multiresonance spectral features to physical fluid properties. This approach exploits the fact that changes in viscosity or density alter the added mass and damping of the resonator, producing measurable variations in the spectral response, including shifts in resonance amplitude and frequency. Machine learning captures these relationships through statistical modeling; however, even studies employing physical models ultimately rely on mathematical approximations of the hydrodynamic function [52,53]. However, in our study, robustness relies on controlled measurement conditions and the proposed drift correction; explicit disentanglement of confounders (e.g., bubbles, mounting stress, aging) is not yet addressed and will be investigated in future work (e.g., additional sensing inputs or physics-informed models).

From a computational perspective, COMSOL simulations could be extended to higher frequencies to account for the evaluation performed without liquid interaction. Moreover, Equation (1) could be refined by incorporating the drift experiment results into the denominator, thereby optimizing another key aspect of sensor performance. Although short-term drift has been characterized, long-term stability studies spanning multiple weeks and multiple devices represent an important next step toward practical deployment. Additional preprocessing and correction techniques may also further improve data quality.

Finally, successful commercialization will require complementing these technical developments with a comprehensive business strategy. Such a strategy should include detailed market analysis, evaluation of regulatory requirements and brand development to ensure the competitiveness and long-term viability of the proposed technology.

## 5. Conclusions

In this work, we introduced a system that integrates 3D-printed fluidic cells and piezoelectric actuators with machine learning algorithms for the monitoring of liquids of industrial and environmental relevance. The transducer was structurally optimized using a novel metric that accounts for the multi-peak characteristics of the electromechanical response, as well as sensitivity, resolution and regeneration capacity. Convolutional neural networks were employed and fine-tuned through hyperparameter optimization to improve accuracy in estimating fluid physical properties from spectral measurements. Both the sensor and the trained networks were implemented within a microcontroller-based platform with simple conditioning electronics. Calibration and resolution errors in viscosity and density estimation were small in comparison with reported values, reaching 0.99% and $1.48\times 10^{-2}$ mPa·s for viscosity, and 0.0485% and $1.9\times 10^{-4}$ g/mL for density. These precision levels enabled the detection of variations in biodiesel composition of less than 1% based on viscosity and density measurements. Additional experiments addressing sensor temporal drift and dimensionality reduction further extended device lifespan and accelerated measurement speed. These results were benchmarked against comparable studies in the literature, demonstrating similar or superior performance in certain cases. While further improvements are possible, particularly through the automation of sensor fabrication, the present work provides effective solutions to challenges related to drift correction, geometric optimization and data dimensionality reduction. The outcome is a low-cost, compact, portable and precise sensing device capable of supporting biofuel quality assessment and adaptable to other industrial applications.

## Figures and Tables

**Table 1:** Description and dimensional specification of the geometric parameters investigated during the sensor optimization process.

Label	Description	Minimum (mm)	Maximum (mm)
$D_{f}^{l}$	X-distance between fins	1	4
$D_{f}^{w}$	Y-distance between fins	0.8	1.82
$H_{f}$	height of the fins	0.9	1.8
$H_{c}$	height of the cavity	1.8	1.8
$L_{f}$	length of the fins	0.5	2
$L_{m}$	length of the membrane	8	17
$L_{p}$	length of the patches	$L_{m}/3+1$	$L_{m}/2+1$
$n_{f}^{l}$	number of fins along X-axis	0	4
$n_{f}^{w}$	number of fins along Y-axis	0	3
$T_{c}$	thickness of the cell	0.2	0.3
$T_{p}$	thickness of the patches	0.11	0.11
$T_{m}$	thickness of the membrane	0.2	0.5
$W_{f}$	width of the fins	0.14	0.6
$W_{m}$	width of the membrane	4	14
$W_{p}$	width of the patches	$W_{m}/2$	$W_{m}$

**Table 2:** Composition of the biofuels employed, with their corresponding viscosity and density values.

Label	Description	η (mPa·s)	ρ (g/mL)
$N_{1}$	100% Diesel-0% Sunflower oil	2.350	0.821
$N_{2}$	95% Diesel-5% Sunflower oil	2.670	0.825
$N_{3}$	90% Diesel-10% Sunflower oil	3.011	0.829
$N_{4}$	85% Diesel-15% Sunflower oil	3.406	0.833
$N_{5}$	80% Diesel-20% Sunflower oil	3.871	0.837
$T_{1}$	99.7% Diesel-0.3% Sunflower oil	2.374	0.821
$T_{2}$	99.5% Diesel-0.5% Sunflower oil	2.381	0.821
$T_{3}$	99% Diesel-1% Sunflower oil	2.410	0.822
$T_{4}$	97.5% Diesel-2.5% Sunflower oil	2.510	0.823
$T_{5}$	87.5% Diesel-12.5% Sunflower oil	3.197	0.831
$T_{6}$	78% Diesel-22% Sunflower oil	4.058	0.839

**Table 3:** General structure of the tested convolutional neural networks.

Input: spectrum of 530 components	
Convolutional blocks ∈ [3, 4]	Number of filters ∈ [8, 64]
	Kernel size ∈ [3, 13]
	Stride ∈ [2, 7]
	ReLU activation function
	MaxPooling, size = 1/2
Average pooling	
Fully connected 1. Output: Viscosity or Density	

**Table 4:** Geometric parameter ranges corresponding to the best-performing designs in terms of sensitivity, resolution, recovery, $m_{1}$ and $m_{2}$. The dimensions of the best sensor found in the experiments are also provided.

Label	Sensitivity	Resolution	Recovery	$m_{1}$	$m_{2}$	Best
$D_{f}^{l}$	1.66–2.33	1–3.5	2.33	1.66–2.33	1–2.33	2.33
$D_{f}^{w}$	1.82	1.3–1.82	1.82	1.82	1.82	1.82
$H_{f}$	0.9	0.9	0.9	0.9	0.9	0.9
$H_{c}$	1.8	1.8	1.8	1.8	1.8	1.8
$L_{f}$	1–2	1–2	1	1–2	0.5–2	1
$L_{m}$	9–14	9	9	9	9	9
$L_{p}$	$L_{m}/2+1$	$L_{m}/2+1$	$L_{m}/2+1$	$L_{m}/2+1$	$L_{m}/2+1$	$L_{m}/2+1$
$n_{f}^{l}$	0–2	2–4	0–2	0–2	0–2	2
$n_{f}^{w}$	0–2	1–3	0–2	0–2	0–2	2
$T_{c}$	0.3	0.2–0.3	0.2–0.3	0.3	0.2–0.3	0.3
$T_{p}$	0.11	0.11	0.11	0.11	0.11	0.11
$T_{m}$	0.5	0.2–0.5	0.2–0.5	0.5	0.2–0.5	0.5
$W_{f}$	0.27	0.14–0.27	0.27	0.27	0.27	0.27
$W_{m}$	6–14	4–6	6	6	6	6
$W_{p}$	$W_{m}/2$	$\frac{W_{m}}{2}-W_{m}$	$\frac{W_{m}}{2}-W_{m}$	$\frac{W_{m}}{2}-W_{m}$	$\frac{W_{m}}{2}-W_{m}$	$W_{m}/2$

**Table 5:** Error values obtained from viscosity predictions using the proposed system.

Dataset	Calibration			Resolution
	e (mPa·s)	e (%FS)	e (%RD)	$e_{\mathrm{res}}$ (mPa·s)
Train (*N*)	$3.46\times 10^{-3}$	0.2	0.12	$2.63\times 10^{-3}$
Test (*T*)	$2.8\times 10^{-2}$	1.64	0.99	$1.48\times 10^{-2}$

**Table 6:** Error values obtained from density predictions using the proposed system.

Dataset	Calibration			Resolution
	e (g/mL)	e (%FS)	e (%RD)	$e_{\mathrm{res}}$ (g/mL)
Train (*N*)	$5.25\times 10^{-5}$	0.29	$6.34\times 10^{-3}$	$4.63\times 10^{-5}$
Test (*T*)	$4\times 10^{-4}$	2.24	$4.85\times 10^{-2}$	$1.9\times 10^{-4}$

**Table 7:** Comparison of viscosity prediction errors between day 0 and subsequent days.

Dataset	Calibration			Resolution
	e (mPa·s)	e (%FS)	e (%RD)	$e_{\mathrm{res}}$ (mPa·s)
Data 0	$1.28\times 10^{-2}$	2.12	0.51	$7.72\times 10^{-3}$
Data drift	$5.5\times 10^{-2}$	9.08	2.04	$2.41\times 10^{-2}$

**Table 8:** Comparison of density prediction errors between day 0 and subsequent days.

Dataset	Calibration			Resolution
	e (g/mL)	e (%FS)	e (%RD)	$e_{\mathrm{res}}$ (g/mL)
Data 0	$1.12\times 10^{-4}$	1.6	$1.36\times 10^{-2}$	$8.09\times 10^{-5}$
Data drift	$5.51\times 10^{-4}$	7.87	$6.7\times 10^{-2}$	$2.03\times 10^{-4}$

**Table 9:** Errors in viscosity estimation for the test dataset using models trained with spectra of varying dimensionality.

Points (Dataset)	Calibration			Resolution
	e (mPa·s)	e (%FS)	e (%RD)	$e_{\mathrm{res}}$ (mPa·s)
530 (*T*)	$2.8\times 10^{-2}$	1.64	0.99	$1.48\times 10^{-2}$
244 (*T*)	$2.31\times 10^{-2}$	1.35	0.84	$2.05\times 10^{-2}$
103 (*T*)	$3.7\times 10^{-2}$	2.18	1.44	$1.52\times 10^{-2}$

**Table 10:** Errors in density estimation for the test dataset using models trained with spectra of varying dimensionality.

Points (Dataset)	Calibration			Resolution
	e (g/mL)	e (%FS)	e (%RD)	$e_{\mathrm{res}}$ (g/mL)
530 (*T*)	$4\times 10^{-4}$	2.24	$4.85\times 10^{-2}$	$1.9\times 10^{-4}$
244 (*T*)	$5.65\times 10^{-4}$	3.14	$6.81\times 10^{-2}$	$2.27\times 10^{-4}$
103 (*T*)	$7.12\times 10^{-4}$	3.95	$8.55\times 10^{-2}$	$1.68\times 10^{-4}$

**Table 11:** Errors in viscosity estimation from the drift experiment using the spectral correction method and models trained with spectra of different numbers of points.

Points (Dataset)	Calibration			Resolution
	e (mPa·s)	e (%FS)	e (%RD)	$e_{\mathrm{res}}$ (mPa·s)
530 (drift)	$6.1\times 10^{-2}$	10.12	2.28	$2.54\times 10^{-2}$
244 (drift)	$5.21\times 10^{-2}$	8.67	1.93	$2.12\times 10^{-2}$
103 (drift)	$6.32\times 10^{-2}$	10.52	2.35	$1.61\times 10^{-2}$

**Table 12:** Errors in density estimation from the drift experiment using the spectral correction method and models trained with spectra of different numbers of points.

Points (Dataset)	Calibration			Resolution
	e (g/mL)	e (%FS)	e (%RD)	$e_{\mathrm{res}}$ (g/mL)
530 (drift)	$5.62\times 10^{-4}$	8.027	$6.8\times 10^{-2}$	$2.18\times 10^{-4}$
244 (drift)	$8.74\times 10^{-4}$	12.48	0.11	$2.16\times 10^{-4}$
103 (drift)	$1.05\times 10^{-3}$	15.03	0.13	$1.97\times 10^{-4}$

**Table 13:** Comparison of viscosity results obtained in this work with those reported in the literature [5,6,7,9,10,17,42,43].

Error	Our Work	[17]	Outperformed by (Excluding [17])
*e* (mPa·s)	$2.8\times 10^{-2}$	$2.1\times 10^{-2}$	-
*e* (%FS)	1.64	1.41	-
*e* (%RD)	0.99	0.8	0.792—[7]
$e_{res}$ (mPa·s)	$1.48\times 10^{-2}$	$1\times 10^{-2}$	[2.7–7.8]$\cdot 10^{-3}$—[6,42,43]

**Table 14:** Comparison of density results obtained in this work with those reported in the literature [5,6,7,17,42,43].

Error	Our Work	[17]	Outperformed by (Excluding [17])
*e* (g/mL)	$4\times 10^{-4}$	$3.5\times 10^{-4}$	$2.3\times 10^{-4}$—[7]
*e* (%FS)	2.24	2.21	1.31—[43]
*e* (%RD)	$4.85\times 10^{-2}$	$4.3\times 10^{-2}$	$2.7\times 10^{-4}$—[7]
$e_{res}$ (g/mL)	$1.9\times 10^{-4}$	$1.33\times 10^{-4}$	[0.24–3.9]$\times 10^{-5}$—[6,7,42]

## Data Availability

The datasets presented in this article are not readily available due to technical/time limitations and because the data are part of an ongoing study. Requests to access the datasets should be directed to victor.corsino@uclm.es.

## References

[1] Razzaq L., Farooq M., Mujtaba M., Sher F., Farhan M., Hassan M.T., Soudagar M.E.M., Atabani A., Kalam M.A., Imran M. (2020). Modeling viscosity and density of ethanol-diesel-biodiesel ternary blends for sustainable environment. Sustainability.

[2] Estevez R., Aguado-Deblas L., Posadillo A., Hurtado B., Bautista F.M., Hidalgo J.M., Luna C., Calero J., Romero A.A., Luna D. (2019). Performance and emission quality assessment in a diesel engine of straight castor and sunflower vegetable oils, in diesel/gasoline/oil triple blends. Energies.

[3] Sun H., Liu Y., Tan J. (2021). Research on testing method of oil characteristic based on quartz tuning fork sensor. Appl. Sci..

[4] Voglhuber-Brunnmaier T., Niedermayer A.O., Feichtinger F., Jakoby B. (2019). Fluid sensing using quartz tuning forks—Measurement technology and applications. Sensors.

[5] Toledo J., Manzaneque T., Hernando-García J., Vázquez J., Ababneh A., Seidel H., Lapuerta M., Sánchez-Rojas J.L. (2014). Application of quartz tuning forks and extensional microresonators for viscosity and density measurements in oil/fuel mixtures. Microsyst. Technol..

[6] Toledo J., Ruiz-Díez V., Pfusterschmied G., Schmid U., Sánchez-Rojas J. (2017). Characterization of oscillator circuits for monitoring the density-viscosity of liquids by means of piezoelectric MEMS microresonators. Proceedings of the Smart Sensors, Actuators, and MEMS VIII.

[7] Toledo J., Manzaneque T., Ruiz-Díez V., Jiménez-Márquez F., Kucera M., Pfusterschmied G., Wistrela E., Schmid U., Sánchez-Rojas J.L. (2016). Comparison of in-plane and out-of-plane piezoelectric microresonators for real-time monitoring of engine oil contamination with diesel. Microsyst. Technol..

[8] Goel S., Venkateswaran P., Prajesh R., Agarwal A. (2015). Rapid and automated measurement of biofuel blending using a microfluidic viscometer. Fuel.

[9] Maurya R.K., Kaur R., Kumar R., Agarwal A. (2019). A novel electronic micro-viscometer. Microsyst. Technol..

[10] Camas-Anzueto J., Gómez-Pérez J., Meza-Gordillo R., Anzueto-Sánchez G., Pérez-Patricio M., López-Estrada F., Abud-Archila M., Ríos-Rojas C. (2017). Measurement of the viscosity of biodiesel by using an optical viscometer. Flow Meas. Instrum..

[11] Chauhan M., Khanikar T., Singh V.K. (2022). PDMS coated fiber optic sensor for efficient detection of fuel adulteration. Appl. Phys. B.

[12] Liu H., Tang X., Lu H., Xie W., Hu Y., Xue Q. (2020). An interdigitated impedance microsensor for detection of moisture content in engine oil. Nanotechnol. Precis. Eng..

[13] Urban A., Zhe J. (2022). A microsensor array for diesel engine lubricant monitoring using deep learning with stochastic global optimization. Sens. Actuators A Phys..

[14] De Pascali C., Bellisario D., Signore M.A., Sciurti E., Radogna A.V., Francioso L.N. (2024). A rapid classification of cross-contaminations in aviation oil using impedance-driven supervised machine learning. IEEE Sens. J..

[15] Moradkhani A., Hasannejad O., Baghelani M. (2022). An artificial intelligence assisted distance variation robust microwave sensor for biofuel analysis applications. IEEE Microw. Wirel. Components Lett..

[16] Baghelani M., Hosseini N., Daneshmand M. (2020). Artificial intelligence assisted noncontact microwave sensor for multivariable biofuel analysis. IEEE Trans. Ind. Electron..

[17] Corsino V., Ruiz-Díez V., Sánchez-Rojas J.L. (2025). Innovative sensor systems using deep learning and piezoelectric resonators for biofuel monitoring. IEEE Sens. Lett..

[18] Košir T., Slavič J. (2022). Single-process fused filament fabrication 3D-printed high-sensitivity dynamic piezoelectric sensor. Addit. Manuf..

[19] Islam S., Tan J.Y., Mou T.D., Ray S., Liu H., Kim J.K., Kim A. (2024). 3D-Printable Self-Powered Piezoelectric Smart Stent for Wireless Endoleaks Sensing. Proceedings of the 2024 IEEE 37th International Conference on Micro Electro Mechanical Systems (MEMS).

[20] Islam M.N., Rupom R.H., Adhikari P.R., Demchuk Z., Popov I., Sokolov A.P., Wu H.F., Advincula R.C., Dahotre N., Jiang Y. (2023). Boosting piezoelectricity by 3D printing PVDF-MoS2 composite as a conformal and high-sensitivity piezoelectric sensor. Adv. Funct. Mater..

[21] Pala S., Lin L. (2019). Fully transparent piezoelectric ultrasonic transducer with 3D printed substrate. Proceedings of the 2019 20th International Conference on Solid-State Sensors, Actuators and Microsystems & Eurosensors XXXIII (TRANSDUCERS & EUROSENSORS XXXIII).

[22] Nassar H., Khandelwal G., Chirila R., Karagiorgis X., Ginesi R.E., Dahiya A.S., Dahiya R. (2023). Fully 3D printed piezoelectric pressure sensor for dynamic tactile sensing. Addit. Manuf..

[23] Feng G.H., Chen W.S. (2022). Piezoelectric micromachined ultrasonic transducer-integrated Helmholtz resonator with microliter-sized volume-tunable cavity. Sensors.

[24] Feng G.H., Chen W.S. (2021). Sound Pressure and Bandwidth Enhanced PMUT with Volume Controllable Helmhotz Resonator for Respiratory Monitoring. Proceedings of the 2021 21st International Conference on Solid-State Sensors, Actuators and Microsystems (Transducers).

[25] Roloff T., Mitkus R., Lion J.N., Sinapius M. (2022). 3D-printable piezoelectric composite sensors for acoustically adapted guided ultrasonic wave detection. Sensors.

[26] Pagliano S., Marschner D.E., Maillard D., Ehrmann N., Stemme G., Braun S., Villanueva L.G., Niklaus F. (2022). Micro 3D printing of a functional MEMS accelerometer. Microsyst. Nanoeng..

[27] Allah A.H., Eyebe G.A., Domingue F. (2022). Fully 3D-printed microfluidic sensor using substrate integrated waveguide technology for liquid permittivity characterization. IEEE Sens. J..

[28] Salim A., Ghosh S., Lim S. (2018). Low-cost and lightweight 3D-printed split-ring resonator for chemical sensing applications. Sensors.

[29] Sebechlebska T., Vaneckova E., Choińska-Młynarczyk M.K., Navrátil T., Poltorak L., Bonini A., Vivaldi F., Kolivoška V. (2022). 3D printed platform for impedimetric sensing of liquids and microfluidic channels. Anal. Chem..

[30] Wiltshire B.D., Zarifi M.H. (2018). 3-D printing microfluidic channels with embedded planar microwave resonators for RFID and liquid detection. IEEE Microw. Wirel. Components Lett..

[31] Wiltshire B.D., Mohammadi S., Zarifi M.H. (2018). Integrating 3D printed microfluidic channels with planar resonator sensors for low cost and sensitive liquid detection. Proceedings of the 2018 18th International Symposium on Antenna Technology and Applied Electromagnetics (ANTEM).

[32] Rocco G.M., Bozzi M., Schreurs D., Perregrini L., Marconi S., Alaimo G., Auricchio F. (2019). 3-D printed microfluidic sensor in SIW technology for liquids’ characterization. IEEE Trans. Microw. Theory Tech..

[33] Moscato S., Pasian M., Bozzi M., Perregrini L., Bahr R., Le T., Tentzeris M.M. (2015). Exploiting 3D printed substrate for microfluidic SIW sensor. Proceedings of the 2015 European microwave conference (EuMC).

[34] Cinar A., Basaran S.C. (2024). 3D-printed sensor design based on metamaterial absorber for characterization of solid and liquid materials. Sens. Actuators Phys..

[35] Mohammed A.M., Hart A., Wood J., Wang Y., Lancaster M.J. (2021). 3D printed re-entrant cavity resonator for complex permittivity measurement of crude oils. Sens. Actuators A Phys..

[36] Zhang D., Wei H., Krishnaswamy S. (2019). 3D printing optofluidic Mach-Zehnder interferometer on a fiber tip for refractive index sensing. IEEE Photonics Technol. Lett..

[37] Radonić V., Birgermajer S., Kitić G. (2017). Microfluidic EBG sensor based on phase-shift method realized using 3D printing technology. Sensors.

[38] Hou Y., Gao M., Gao J., Zhao L., Teo E.H.T., Wang D., Qi H.J., Zhou K. (2023). 3D printed conformal strain and humidity sensors for human motion prediction and health monitoring via machine learning. Adv. Sci..

[39] Liu G., Wang C., Jia Z., Wang K., Ma W., Li Z. (2021). A rapid design and fabrication method for a capacitive accelerometer based on machine learning and 3D printing techniques. IEEE Sens. J..

[40] Liu G., Yu P., Tao Y., Liu T., Liu H., Zhao J. (2024). Hybrid 3D printed three-axis force sensor aided by machine learning decoupling. Int. J. Smart Nano Mater..

[41] Song Y., Tay R.Y., Li J., Xu C., Min J., Shirzaei Sani E., Kim G., Heng W., Kim I., Gao W. (2023). 3D-printed epifluidic electronic skin for machine learning–powered multimodal health surveillance. Sci. Adv..

[42] Toledo J., Ruiz-Díez V., Velasco J., Hernando-García J., Sánchez-Rojas J.L. (2021). 3D-printed liquid cell resonator with piezoelectric actuation for in-line density-viscosity measurements. Sensors.

[43] Corsino V., Ruiz-Díez V., Gilpérez J.M., Ramírez-Palma M., Sánchez-Rojas J.L. (2023). Machine learning techniques for the estimation of viscosity and density of aqueous solutions in piezo-actuated 3D-printed cells. Sens. Actuators A Phys..

[44] Formlabs Formlabs Resin 3D Printer. https://formlabs.com/.

[45] Formlabs Rigid 10K Resin Datasheet. https://formlabs-media.formlabs.com/datasheets/2001479-TDS-ENUS-0.pdf.

[46] Diener Electronic FEMTO. Low-Pressure Plasma Systems (Plasmacleaner). https://www.plasma.com/en/low-pressureplasma-femto/.

[47] PI-Ceramic PZT Actuators. https://www.piceramic.com/en/products/piezoceramic-components/plates-and-blocks/.

[48] Henkel Loctite 435. https://www.henkel-adhesives.com/es/es/producto/instant-adhesives/loctite_4350.html.

[49] Veeco VEECO Wyko NT1100 Optical Profiler. https://www.veeco.com/.

[50] Theisen L.A., Martin S.J., Hillman A.R. (2004). A model for the quartz crystal microbalance frequency response to wetting characteristics of corrugated surfaces. Anal. Chem..

[51] Keysight 4294A Precision Impedance Analyzer, 40 Hz to 110 MHz. https://www.keysight.com/us/en/support/4294A/precision-impedance-analyzer-40-hz-to-110-mhz.html.

[52] Sader J.E. (1998). Frequency response of cantilever beams immersed in viscous fluids with applications to the atomic force microscope. J. Appl. Phys..

[53] Dufour I., Lemaire E., Caillard B., Debéda H., Lucat C., Heinrich S.M., Josse F., Brand O. (2014). Effect of hydrodynamic force on microcantilever vibrations: Applications to liquid-phase chemical sensing. Sens. Actuators B Chem..

[54] COMSOL Software for Multiphysics Simulation. https://www.comsol.com/.

[55] Toledo J., Ruiz-Díez V., Díaz A., Ruiz D., Donoso A., Bellido J.C., Wistrela E., Kucera M., Schmid U., Hernando-García J. (2017). Design and characterization of in-plane piezoelectric microactuators. Actuators.

[56] Corsino V., Ruiz-Díez V., Sánchez-Rojas J.L. (2025). Smart density and viscosity sensing based on edge machine learning and piezoelectric MEMS for edible oil monitoring. Sens. Actuators A Phys..

[57] Digilent Analog Discovery 2. https://digilent.com/reference/test-and-measurement/analog-discovery-2/start?srsltid=AfmBOoqzO4NnlfHMxz82_WnP0s9Fm64nW8wV5yEbcaJcbfjwHyum5WkA.

[58] Systems E. ESP32-S3-DevKitC-1-N32R8V. https://docs.espressif.com/projects/esp-idf/en/latest/esp32s3/hw-reference/esp32s3/user-guide-devkitc-1.html.

[59] Gilson Gilson Peristaltic Pump Minipuls 3. https://es.gilson.com/minipuls-3-peristaltic-pumps.html.

[60] Paar A. Rolling-Ball Viscometer: Lovis 2000 M/ME. https://www.anton-paar.com/corp-en/products/details/rolling-ball-viscometer-lovis-2000-mme/.

[61] Kingma D.P., Ba J. (2014). Adam: A method for stochastic optimization. arXiv.

[62] Abadi M., Agarwal A., Barham P., Brevdo E., Chen Z., Citro C., Corrado G.S., Davis A., Dean J., Devin M. TensorFlow: Large-Scale Machine Learning on Heterogeneous Systems, 2015. https://www.tensorflow.org/.

[63] Yu Y., Zheng T., Zhang N., Wu J. (2022). Review of sintering aids in lead-free (K, Na) NbO_3_-based ceramics. IEEE Trans. Ultrason. Ferroelectr. Freq. Control.

[64] Wu Y., Wang G., Jiao Z., Fan Y., Peng P., Dong X. (2019). High electrostrictive properties and energy storage performances with excellent thermal stability in Nb-doped Bi_0.5_Na_0.5_TiO_3_-based ceramics. RSC Adv..

